# Supplementary material for: Overview of Risk Factors for Esophageal Squamous Cell Carcinoma in China
Source: Cancers (Basel). 2023 Nov 27;15(23):5604. doi: 10.3390/cancers15235604 (PMC10705141; doi:10.3390/cancers15235604)
Supplement: Supplementary file 1 [file cancers-15-05604-s001.zip › Supplementary Materials- References for included studies (207).pdf]

1. Gu, H.; Wang, X.; Zheng, L.; Tang, W.; Dong, C.; Wang, L.; Shi, Y.; Shao, A.; Ding, G.; Liu, C.; et al. Vitamin D Receptor Gene Polymorphisms and Esophageal Cancer Risk in a Chinese Population: A Negative Study. *Med Oncol* **2014**, *31*, 827, doi:10.1007/s12032-013-0827-x.
2. Shen, F.F.; Yue, W.B.; Zhou, F.Y.; Pan, Y.; Zhao, X.K.; Jin, Y.; Song, X.; Li, B.; Han, X.N.; Tang, S.; et al. Variations in the MHC Region Confer Risk to Esophageal Squamous Cell Carcinoma on the Subjects from High-Incidence Area in Northern China. *PLoS One* **2014**, *9*, e90438, doi:10.1371/journal.pone.0090438.
3. Yin, J.; Sang, Y.; Zheng, L.; Wang, L.; Yuan, L.; Liu, C.; Wang, X.; Shi, Y.; Shao, A.; Ding, G.; et al. Uracil-DNA Glycosylase (UNG) Rs246079 G/A Polymorphism Is Associated with Decreased Risk of Esophageal Cancer in a Chinese Population. *Medical Oncology* **2014**, *31*, 1–8, doi:10.1007/s12032-014-0272-5.
4. Du, J.; Xue, W.; Ji, Y.; Zhu, X.; Gu, Y.; Zhu, M.; Wang, C.; Gao, Y.; Dai, J.; Ma, H.; et al. U-Shaped Association between Telomere Length and Esophageal Squamous Cell Carcinoma Risk: A Case-Control Study in Chinese Population. *Frontiers of medicine* **2015**, *9*, 478–486, doi:10.1007/s11684-015-0420-0.
5. Chang, J.; Wei, L.; Miao, X.; Yu, D.; Tan, W.; Zhang, X.; Wu, C.; Lin, D. Two Novel Variants on 13q22.1 Are Associated with Risk of Esophageal Squamous Cell Carcinoma. *Cancer Epidemiol Biomarkers Prev* **2015**, *24*, 1774–1780, doi:10.1158/1055-9965.Epi-15-0154-t.
6. Shi, M.; Xia, J.; Xing, H.; Yang, W.; Xiong, X.; Pan, W.; Han, S.; Shang, J.; Zhou, C.; Zhou, L.; et al. The Sp1-Mediaded Allelic Regulation of MMP13 Expression by an ESCC Susceptibility SNP Rs2252070. *Scientific reports* **2016**, *6*, 27013, doi:10.1038/srep27013.
7. Su, F.; Fang, Y.; Yu, J.; Jiang, T.; Lin, S.; Zhang, S.; Lv, L.; Long, T.; Pan, H.; Qi, J.; et al. The Single Nucleotide Polymorphisms of AP1S1 Are Associated with Risk of Esophageal Squamous Cell Carcinoma in Chinese Population. *Pharmacogenomics and Personalized Medicine* **2022**, *15*, 235–247, doi:10.2147/PGPM.S342743.
8. Jiang, L.; Wang, C.; Sun, C.; Xu, Y.; Ding, Z.; Zhang, X.; Huang, J.; Yu, H. The Impact of Pri-MiR-218 Rs11134527 on the Risk and Prognosis of Patients with Esophageal Squamous Cell Carcinoma. *International journal of clinical and experimental pathology* **2014**, *7*, 6206–6212.
9. Zhou, L.; Fu, G.; Wei, J.; Shi, J.; Pan, W.; Ren, Y.; Xiong, X.; Xia, J.; Shen, Y.; Li, H.; et al. The Identification of Two Regulatory ESCC Susceptibility Genetic Variants in the TERT-CLPTM1L Loci. *Oncotarget* **2016**, *7*, 5495–5506, doi:10.18632/oncotarget.6747.
10. Zhang, X.; Zhou, L.; Fu, G.; Sun, F.; Shi, J.; Wei, J.; Lu, C.; Zhou, C.; Yuan, Q.; Yang, M. The Identification of an ESCC Susceptibility SNP Rs920778 That Regulates the Expression of LncRNA HOTAIR via a Novel Intronic Enhancer. *Carcinogenesis* **2014**, *35*, 2062–2067, doi:10.1093/carcin/bgu103.
11. Yao, Y.; Shao, J.; Wu, J.; Zhang, Q.; Wang, J.; Xiao, D.; Huang, F. The Functional Variant in the 3'UTR of PTPRT with the Risk of Esophageal Squamous Cell Carcinoma in a Chinese Population. *Cellular Physiology and Biochemistry* **2015**, *36*, 306–314, doi:10.1159/000374073.
12. Peng, H.; Wang, L.G.; Wang, X.Z.; Liu, A.J. The Correlation between XIAP Gene Polymorphisms and Esophageal Squamous Cell Carcinoma Susceptibility and Prognosis in a Chinese Population. *Pathology Research and Practice* **2017**, *213*, 1482–1488, doi:10.1016/j.prp.2017.10.008.
13. Yin, J.; Wang, L.; Zheng, L.; Wang, X.; Shi, Y.; Shao, A.; Ding, G.; Liu, C.; Chen, S.; Tang, W.; et al. TERT-CLPTM1L Rs401681 C>T Polymorphism Was Associated with a Decreased Risk of Esophageal Cancer in a Chinese Population. *PLoS One* **2014**, *9*, e100667, doi:10.1371/journal.pone.0100667.
14. Chen, P.; Song, Q.; Han, J.; Xu, H.; Chen, T.; Xu, J.; Cheng, Y. Sitting Time and Occupational and Recreational Physical Activity in Relation to the Risk of Esophageal Squamous Cell Carcinoma. *OncoTargets and Therapy* **2017**, *10*, 4787–4794, doi:10.2147/OTT.S147711.
15. Qu, Y.; Zhang, Y.; Wang, K.; Song, C.; Wang, P.; Ye, H.; Zhang, J.; Dai, L. Single Nucleotide Polymorphisms in MicroRNA-Binding Site of Epidermal Growth Factor Receptor Signaling Pathway and Susceptibility to Esophageal Squamous Cell Carcinoma. *Dig Dis* **2020**, *38*, 1–8, doi:10.1159/000501447.
16. Yue, C.M.; Bi, M.X.; Tan, W.; Deng, D.J.; Zhang, X.Y.; Guo, L.P.; Lin, D.X.; Lu, S.H. Short Tandem Repeat Polymorphism in a Novel Esophageal Cancer-Related Gene (ECRG2) Implicates Susceptibility to Esophageal Cancer in Chinese Population. *Int J Cancer* **2004**, *108*, 232–236, doi:10.1002/ijc.11560.
17. Lin, S.W.; Fan, J.H.; Dawsey, S.M.; Taylor, P.R.; Qiao, Y.L.; Abnet, C.C. Serum Thyroglobulin, a Biomarker for Iodine Deficiency, Is Not Associated with Increased Risk of Upper Gastrointestinal Cancers in a Large Chinese Cohort. *Int J Cancer* **2011**, *129*, 2284–2289, doi:10.1002/ijc.25789.
18. Wang, J.; Wei, J.; Xu, X.; Pan, W.; Ge, Y.; Zhou, C.; Liu, C.; Gao, J.; Yang, M.; Mao, W. Replication Study of ESCC Susceptibility Genetic Polymorphisms Locating in the ADH1B-ADH1C-ADH7 Cluster Identified by GWAS. *PLoS ONE* **2014**, *9*, e94096, doi:10.1371/journal.pone.0094096.

19. Ng, D.; Hu, N.; Hu, Y.; Wang, C.; Giffen, C.; Tang, Z.Z.; Han, X.Y.; Yang, H.H.; Lee, M.P.; Goldstein, A.M.; et al. Replication of a Genome-Wide Case-Control Study of Esophageal Squamous Cell Carcinoma. *Int J Cancer* **2008**, *123*, 1610–1615, doi:10.1002/ijc.23682.
20. Hu, H.C.; Yang, J.M.; Sun, Y.H.; Yang, Y.J.; Qian, J.; Jin, L.; Wang, M.Y.; Bi, R.; Zhang, R.X.; Zhu, M.L.; et al. Putatively Functional PLCE1 Variants and Susceptibility to Esophageal Squamous Cell Carcinoma (ESCC): A Case-Control Study in Eastern Chinese Populations. *Annals of Surgical Oncology* **2012**, *19*, 2403–2410, doi:10.1245/s10434-011-2160-y.
21. Ge, H.; Cao, Y.Y.; Chen, L.Q.; Wang, Y.M.; Chen, Z.F.; Wen, D.G.; Zhang, X.F.; Guo, W.; Wang, N.; Li, Y.; et al. PTEN Polymorphisms and the Risk of Esophageal Carcinoma and Gastric Cardiac Carcinoma in a High Incidence Region of China. *Diseases of the Esophagus* **2008**, *21*, 409–415, doi:10.1111/j.1442-2050.2007.00786.x.
22. Murphy, G.; Fan, J.H.; Mark, S.D.; Dawsey, S.M.; Selhub, J.; Wang, J.; Taylor, P.R.; Qiao, Y.L.; Abnet, C.C. Prospective Study of Serum Cysteine Levels and Oesophageal and Gastric Cancers in China. *Gut* **2011**, *60*, 618–623, doi:10.1136/gut.2010.225854.
23. Li, C.L.; Zhao, J.Q.; Zang, B. PRKAA1 Rs13361707 C/T Polymorphism Confers Decreased Susceptibility to Esophageal Cancer: A Case-Control Study. *Journal of Clinical Laboratory Analysis* **2020**, *34*, e23406, doi:10.1002/jcla.23406.
24. Zhang, J.; Huang, X.; Xiao, J.; Yang, Y.; Zhou, Y.; Wang, X.; Liu, Q.; Yang, J.; Wang, M.; Qiu, L.; et al. Pri-MiR-124 Rs531564 and Pri-MiR-34b/c Rs4938723 Polymorphisms Are Associated with Decreased Risk of Esophageal Squamous Cell Carcinoma in Chinese Populations. *PLoS One* **2014**, *9*, e100055, doi:10.1371/journal.pone.0100055.
25. Lam, T.K.; Freedman, N.D.; Fan, J.H.; Qiao, Y.L.; Dawsey, S.M.; Taylor, P.R.; Abnet, C.C. Prediagnostic Plasma Vitamin C and Risk of Gastric Adenocarcinoma and Esophageal Squamous Cell Carcinoma in a Chinese Population. *Am J Clin Nutr* **2013**, *98*, 1289–1297, doi:10.3945/ajcn.113.061267.
26. Yang, X.; Xiong, G.; Chen, X.; Xu, X.; Wang, K.; Fu, Y.; Yang, K.; Bai, Y. Polymorphisms of Survivin Promoter Are Associated with Risk of Esophageal Squamous Cell Carcinoma. *Journal of Cancer Research and Clinical Oncology* **2009**, *135*, 1341–1349, doi:10.1007/s00432-009-0575-7.
27. Xing, D.; Qi, J.; Miao, X.; Lu, W.; Tan, W.; Lin, D. Polymorphisms of DNA Repair Genes XRCC1 and XPD and Their Associations with Risk of Esophageal Squamous Cell Carcinoma in a Chinese Population. *Int J Cancer* **2002**, *100*, 600–605, doi:10.1002/ijc.10528.
28. Xiong, G.; Guo, H.; Wang, K.; Hu, H.; Wang, D.; Xu, X.; Guan, X.; Yang, K.; Bai, Y. Polymorphisms of Decoy Receptor 3 Are Associated with Risk of Esophageal Squamous Cell Carcinoma in Chinese Han. *Tumour Biol* **2010**, *31*, 443–449, doi:10.1007/s13277-010-0053-5.
29. Sun, T.; Miao, X.; Zhang, X.; Tan, W.; Xiong, P.; Lin, D. Polymorphisms of Death Pathway Genes FAS and FASL in Esophageal Squamous-Cell Carcinoma. *J Natl Cancer Inst* **2004**, *96*, 1030–1036, doi:10.1093/jnci/djh187.
30. Zhu, M.L.; Shi, T.Y.; Hu, H.C.; He, J.; Wang, M.; Jin, L.; Yang, Y.J.; Wang, J.C.; Sun, M.H.; Chen, H.; et al. Polymorphisms in the ERCC5 Gene and Risk of Esophageal Squamous Cell Carcinoma (ESCC) in Eastern Chinese Populations. *PLoS One* **2012**, *7*, e41500, doi:10.1371/journal.pone.0041500.
31. Yu, H.P.; Wang, X.L.; Sun, X.; Su, Y.H.; Wang, Y.J.; Lu, B.; Shi, L.Y.; Xiong, C.L.; Li, Y.Y.; Li, F.; et al. Polymorphisms in the DNA Repair Gene XPD and Susceptibility to Esophageal Squamous Cell Carcinoma. *Cancer Genetics and Cytogenetics* **2004**, *154*, 10–15, doi:10.1016/j.cancergencyto.2004.01.027.
32. Zhu, J.; Wang, M.; He, J.; Zhu, M.; Wang, J.C.; Jin, L.; Wang, X.F.; Yang, Y.J.; Xiang, J.Q.; Wei, Q. Polymorphisms in the AKT1 and AKT2 Genes and Oesophageal Squamous Cell Carcinoma Risk in an Eastern Chinese Population. *Journal of Cellular and Molecular Medicine* **2016**, *20*, 666–677, doi:10.1111/jcmm.12750.
33. Zhu, M.L.; Yu, H.; Shi, T.Y.; He, J.; Wang, M.Y.; Li, Q.X.; Sun, M.H.; Jin, L.; Yang, Y.J.; Wang, J.C.; et al. Polymorphisms in MTORC1 Genes Modulate Risk of Esophageal Squamous Cell Carcinoma in Eastern Chinese Populations. *J Thorac Oncol* **2013**, *8*, 788–795, doi:10.1097/JTO.0b013e31828916c6.
34. Zhou, S.L.; Cui, J.; Fan, Z.M.; Li, X.M.; Li, J.L.; Liu, B.C.; Zhang, D.Y.; Liu, H.Y.; Zhao, X.K.; Song, X.; et al. Polymorphism of A133S and Promoter Hypermethylation in Ras Association Domain Family 1A Gene (RASSF1A) Is Associated with Risk of Esophageal and Gastric Cardia Cancers in Chinese Population from High Incidence Area in Northern China. *BMC Cancer* **2013**, *13*, 259, doi:10.1186/1471-2407-13-259.
35. Guo, Y.; Zhang, X.; Tan, W.; Miao, X.; Sun, T.; Zhao, D.; Lin, D. Platelet 12-Lipoxygenase Arg261Gln Polymorphism: Functional Characterization and Association with Risk of Esophageal Squamous Cell Carcinoma in Combination with COX-2 Polymorphisms. *Pharmacogenetics and Genomics* **2007**, *17*, 197–205, doi:10.1097/FPC.0b013e328010bda1.
36. Si, H.X.; Tsao, S.W.; Poon, C.S.P.; Wong, Y.C.; Cheung, A.L.M. Physical Status of HPV-16 in Esophageal Squamous Cell Carcinoma. *Journal of Clinical Virology* **2005**, *32*, 19–23, doi:10.1016/j.jcv.2004.04.004.

37. Duan, F.J.; Xie, W.; Cui, L.H.; Wang, P.; Song, C.H.; Qu, H.H.; Wang, K.J.; Zhang, J.Y.; Dai, L.P. Novel Functional Variants Locus in PLCE1 and Susceptibility to Esophageal Squamous Cell Carcinoma: Based on Published Genome-Wide Association Studies in a Central Chinese Population. *Cancer Epidemiology* **2013**, *37*, 647–652, doi:10.1016/j.canep.2013.04.009.
38. Jin, X.; Kuang, G.; Wei, L.Z.; Li, Y.; Wang, R.; Guo, W.; Wang, N.; Fang, S.M.; Wen, D.G.; Chen, Z.F.; et al. No Association of the Matrix Metalloproteinase 1 Promoter Polymorphism with Susceptibility to Esophageal Squamous Cell Carcinoma and Gastric Cardiac Adenocarcinoma in Northern China. *World J Gastroenterol* **2005**, *11*, 2385–2389, doi:10.3748/wjg.v11.i16.2385.
39. Cao, W.; Chen, X.F.; Dai, H.F.; Shen, B.H.; Chu, D.; McAfee, T.; Zhang, Z.F. Mutational Spectra of P53 in Geographically Localized Esophageal Squamous Cell Carcinoma Groups in China. *Cancer* **2004**, *101*, 834–844, doi:10.1002/cncr.20437.
40. Moody, S.; Senkin, S.; Islam, S.M.A.; Wang, J.; Nasrollahzadeh, D.; Cortez Cardoso Penha, R.; Fitzgerald, S.; Bergstrom, E.N.; Atkins, J.; He, Y.; et al. Mutational Signatures in Esophageal Squamous Cell Carcinoma from Eight Countries with Varying Incidence. *Nat Genet* **2021**, *53*, 1553–1563, doi:10.1038/s41588-021-00928-6.
41. Wei, J.; Zheng, L.; Liu, S.; Yin, J.; Wang, L.; Wang, X.; Shi, Y.; Shao, A.; Tang, W.; Ding, G.; et al. MiR-196a2 Rs11614913 T>C Polymorphism and Risk of Esophageal Cancer in a Chinese Population. *Human Immunology* **2013**, *74*, 1199–1205, doi:10.1016/j.humimm.2013.06.012.
42. Xiao, F.K.; Guo, S.; Yang, F.; Zhao, L.S.; Wang, L.D. MDM2 and Its Functional Polymorphism SNP309 Contribute to the Development of Esophageal Carcinoma. *Journal of Gene Medicine* **2019**, *21*, e3086, doi:10.1002/jgm.3086.
43. Guan, X.; Wang, X.; Luo, H.; Wu, J.; Zhang, X.; Wu, J. Matrix Metalloproteinase 1, 3, and 9 Polymorphisms and Esophageal Squamous Cell Carcinoma Risk. *Med Sci Monit* **2014**, *20*, 2269–2274, doi:10.12659/msm.892413.
44. Xing, E.P.; Yang, G.Y.; Wang, L.D.; Shi, S.T.; Yang, C.S. Loss of Heterozygosity of the Rb Gene Correlates with PRb Protein Expression and Associates with P53 Alteration in Human Esophageal Cancer. *Clin Cancer Res* **1999**, *5*, 1231–1240.
45. Cao, B.; Tian, X.; Li, Y.; Jiang, P.; Ning, T.; Xing, H.; Zhao, Y.; Zhang, C.; Shi, X.; Chen, D.; et al. LMP7/TAP2 Gene Polymorphisms and HPV Infection in Esophageal Carcinoma Patients from a High Incidence Area in China. *Carcinogenesis* **2005**, *26*, 1280–1284, doi:10.1093/carcin/bgi071.
46. Chen, S.; Cao, R.; Liu, C.; Tang, W.; Kang, M. Investigation of IL-4, IL-10, and HVEM Polymorphisms with Esophageal Squamous Cell Carcinoma: A Case-Control Study Involving 1,929 Participants. *Bioscience reports* **2020**, doi:10.1042/BSR20193895.
47. Wei, Y.S.; Lan, Y.; Liu, Y.G.; Tang, H.; Tang, R.G.; Wang, J.C. Interleukin-18 Gene Promoter Polymorphisms and the Risk of Esophageal Squamous Cell Carcinoma. *Acta Oncologica* **2007**, *46*, 1090–1096, doi:10.1080/02841860701373595.
48. Yin, J.; Wang, L.; Shi, Y.; Shao, A.; Tang, W.; Wang, X.; Ding, G.; Liu, C.; Chen, S.; Gu, H. Interleukin 17A Rs4711998 A>G Polymorphism Was Associated with a Decreased Risk of Esophageal Cancer in a Chinese Population. *Diseases of the Esophagus* **2014**, *27*, 87–92, doi:10.1111/dote.12045.
49. Sun, J.M.; Li, Q.; Gu, H.Y.; Chen, Y.J.; Wei, J.S.; Zhu, Q.; Chen, L. Interleukin 10 Rs1800872 T>G Polymorphism Was Associated with an Increased Risk of Esophageal Cancer in a Chinese Population. *Asian Pacific Journal of Cancer Prevention* **2013**, *14*, 3443–3447, doi:10.7314/APJCP.2013.14.6.3443.
50. Yang, H.P.; Liu, J.F.; Rao, J.; Zhang, X.M.; Qian, H.L.; Niu, X.Q.; Zhao, Z.L. Insulin-like Growth Factor Binding Protein-3 (IGFBP-3) Genetic Variant and the Risk of Esophageal Squamous Cell Carcinoma in a Chinese Population. *Genet Mol Res* **2014**, *13*, 4146–4153, doi:10.4238/2014.May.30.10.
51. Xu, X.; Wang, J.; Zhu, S.M.; Yang, M.; Fang, Y.; Zhao, A.; Song, Q.; Mao, W. Impact of Alcohol Dehydrogenase Gene 4 Polymorphisms on Esophageal Squamous Cell Carcinoma Risk in a Chinese Population. *PLoS One* **2015**, *10*, e0127304, doi:10.1371/journal.pone.0127304.
52. Yin, J.; Wang, L.; Shi, Y.; Shao, A.; Tang, W.; Wang, X.; Zhang, W.; Ding, G.; Liu, C.; Chen, Y.; et al. IL-15 Receptor Alpha Rs2228059 A>C Polymorphism Was Associated with a Decreased Risk of Esophageal Cancer in a Chinese Population. *Mol Biol Rep* **2014**, *41*, 1951–1957, doi:10.1007/s11033-014-3042-8.
53. Liu, C.; Tang, W.; Chen, S.; Wang, Y.; Qiu, H.; Yin, J.; Gu, H. IGFBP3 Polymorphisms and Risk of Esophageal Cancer in a Chinese Population. *International Journal of Clinical and Experimental Medicine* **2015**, *8*, 17006–17014.
54. Hu, N.; Roth, M.J.; Polymeropolous, M.; Tang, Z.Z.; Emmert-Buck, M.R.; Wang, Q.H.; Goldstein, A.M.; Feng, S.S.; Dawsey, S.M.; Ding, T.; et al. Identification of Novel Regions of Allelic Loss from a Genomewide Scan of Esophageal Squamous-Cell Carcinoma in a High-Risk Chinese Population. *Genes Chromosomes Cancer* **2000**, *27*, 217–228, doi:10.1002/(sici)1098-2264(200003)27:3<217::aid-gcc1>3.0.co;2-a.
55. Song, Y.; Li, L.; Ou, Y.; Gao, Z.; Li, E.; Li, X.; Zhang, W.; Wang, J.; Xu, L.; Zhou, Y.; et al. Identification of Genomic Alterations in Oesophageal Squamous Cell Cancer. *Nature* **2014**, *509*, 91–95, doi:10.1038/nature13176.

56. Li, Y.; Zhang, X.; Huang, G.; Miao, X.; Guo, L.; Lin, D.; Lu, S.H. Identification of a Novel Polymorphism Arg290Gln of Esophageal Cancer Related Gene 1 (ECRG1) and Its Related Risk to Esophageal Squamous Cell Carcinoma. *Carcinogenesis* **2006**, *27*, 798–802, doi:10.1093/carcin/bgi258.
57. Tang, W.Y.; Wang, L.; Li, C.; Hu, Z.B.; Chen, R.; Zhu, Y.J.; Shen, H.B.; Wei, Q.Y.; Zhou, J.W. Identification and Functional Characterization of JWA Polymorphisms and Their Association with Risk of Gastric Cancer and Esophageal Squamous Cell Carcinoma in a Chinese Population. *J Toxicol Environ Health A* **2007**, *70*, 885–894, doi:10.1080/15287390701285915.
58. Si, H.X.; Tsao, S.W.; Poon, C.S.P.; Cheung, A.L.M. Human Papillomavirus Infection and Loss of Heterozygosity in Esophageal Squamous Cell Carcinoma. *Cancer Letters* **2004**, *213*, 231–239, doi:10.1016/j.canlet.2004.04.008.
59. Ding, G.C.; Ren, J.L.; Chang, F.B.; Li, J.L.; Yuan, L.; Song, X.; Zhou, S.L.; Guo, T.; Fan, Z.M.; Zeng, Y.; et al. Human Papillomavirus DNA and P16INK4A Expression in Concurrent Esophageal and Gastric Cardia Cancers. *World Journal of Gastroenterology* **2010**, *16*, 5901–5906, doi:10.3748/wjg.v16.i46.5901.
60. Yin, J.; Wang, X.; Zheng, L.; Shi, Y.; Wang, L.; Shao, A.; Tang, W.; Ding, G.; Liu, C.; Liu, R.; et al. Hsa-MiR-34b/c Rs4938723 T>C and Hsa-MiR-423 Rs6505162 C>A Polymorphisms Are Associated with the Risk of Esophageal Cancer in a Chinese Population. *PLoS ONE* **2013**, *8*, e80570, doi:10.1371/journal.pone.0080570.
61. Huang, J.; Hu, N.; Goldstein, A.M.; Emmert-Buck, M.R.; Tang, Z.Z.; Roth, M.J.; Wang, Q.H.; Dawsey, S.M.; Han, X.Y.; Ding, T.; et al. High Frequency Allelic Loss on Chromosome 17p13.3-P11.1 in Esophageal Squamous Cell Carcinomas from a High Incidence Area in Northern China. *Carcinogenesis* **2000**, *21*, 2019–2026, doi:10.1093/carcin/21.11.2019.
62. Wang, L.D.; Zhou, F.Y.; Sun, L.D.; Song, X.; Jin, Y.; Li, J.M.; Kong, G.Q.; Qi, H.; Cui, J.; Zhang, L.Q.; et al. Genome-Wide Association Study of Esophageal Squamous Cell Carcinoma in Chinese Subjects Identifies Susceptibility Loci at PLCE1 and C20orf54. *Nature genetics* **2010**, *42*, 759–763, doi:10.1038/ng.648.
63. Wu, C.; Hu, Z.; He, Z.; Jia, W.; Wang, F.; Zhou, Y.; Liu, Z.; Zhan, Q.; Liu, Y.; Yu, D.; et al. Genome-Wide Association Study Identifies Three New Susceptibility Loci for Esophageal Squamous-Cell Carcinoma in Chinese Populations. *Nat Genet* **2011**, *43*, 679–684, doi:10.1038/ng.849.
64. Wu, C.; Kraft, P.; Zhai, K.; Chang, J.; Wang, Z.; Li, Y.; Hu, Z.; He, Z.; Jia, W.; Abnet, C.C.; et al. Genome-Wide Association Analyses of Esophageal Squamous Cell Carcinoma in Chinese Identify Multiple Susceptibility Loci and Gene-Environment Interactions. *Nat Genet* **2012**, *44*, 1090–1097, doi:10.1038/ng.2411.
65. Tang, W.; Zhang, S.; Qiu, H.; Wang, L.; Sun, B.; Yin, J.; Gu, H. Genetic Variations in MTHFR and Esophageal Squamous Cell Carcinoma Susceptibility in Chinese Han Population. *Med Oncol* **2014**, *31*, 915, doi:10.1007/s12032-014-0915-6.
66. Wei, Y.S.; Xu, Q.Q.; Wang, C.F.; Pan, Y.; Liang, F.; Long, X.K. Genetic Variation in Transforming Growth Factor-Beta1 Gene Associated with Increased Risk of Esophageal Squamous Cell Carcinoma. *Tissue Antigens* **2007**, *70*, 464–469, doi:10.1111/j.1399-0039.2007.00935.x.
67. Zhu, J.; Yang, L.; You, W.; Cui, X.; Chen, Y.; Hu, J.; Liu, W.; Li, S.; Song, X.; Wei, Y.; et al. Genetic Variation in MiR-100 Rs1834306 Is Associated with Decreased Risk for Esophageal Squamous Cell Carcinoma in Kazakh Patients in Northwest China. *International journal of clinical and experimental pathology* **2015**, *8*, 7332–7340.
68. Zhang, W.; Chen, X.; Luo, A.; Lin, D.; Tan, W.; Liu, Z. Genetic Variants of C1orf10 and Risk of Esophageal Squamous Cell Carcinoma in a Chinese Population. *Cancer science* **2009**, *100*, 1695–1700, doi:10.1111/j.1349-7006.2009.01240.x.
69. Hyland, P.L.; Freedman, N.D.; Hu, N.; Tang, Z.Z.; Wang, L.; Wang, C.; Ding, T.; Fan, J.H.; Qiao, Y.L.; Golozar, A.; et al. Genetic Variants in Sex Hormone Metabolic Pathway Genes and Risk of Esophageal Squamous Cell Carcinoma. *Carcinogenesis* **2013**, *34*, 1062–1068, doi:10.1093/carcin/bgt030.
70. Shen, F.; Chen, J.; Guo, S.; Zhou, Y.; Zheng, Y.; Yang, Y.; Zhang, J.; Wang, X.; Wang, C.; Zhao, D.; et al. Genetic Variants in MiR-196a2 and MiR-499 Are Associated with Susceptibility to Esophageal Squamous Cell Carcinoma in Chinese Han Population. *Tumor Biology* **2016**, *37*, 4777–4784, doi:10.1007/s13277-015-4268-3.
71. Lin, X.; Yan, C.; Gao, Y.; Du, J.; Zhu, X.; Yu, F.; Huang, T.; Dai, J.; Ma, H.; Jiang, Y.; et al. Genetic Variants at 9p21.3 Are Associated with Risk of Esophageal Squamous Cell Carcinoma in a Chinese Population. *Cancer Sci* **2017**, *108*, 250–255, doi:10.1111/cas.13130.
72. Dai, N.; Zheng, M.; Wang, C.; Ji, Y.; Du, J.; Zhu, C.; He, Y.; Zhu, M.; Zhu, X.; Sun, M.; et al. Genetic Variants at 8q24 Are Associated with Risk of Esophageal Squamous Cell Carcinoma in a Chinese Population. *Cancer Science* **2014**, *105*, 731–735, doi:10.1111/cas.12399.
73. Zhang, Z.; Yu, X.; Guo, Y.; Song, W.; Yu, D.; Zhang, X. Genetic Variant in CASP3 Affects Promoter Activity and Risk of Esophageal Squamous Cell Carcinoma. *Cancer Sci* **2012**, *103*, 555–560, doi:10.1111/j.1349-7006.2011.02173.x.

74. Yang, L.; Ji, Y.; Chen, L.; Li, M.; Wu, F.; Hu, J.; Jiang, J.; Cui, X.; Chen, Y.; Pang, L.; et al. Genetic Variability in LMP2 and LMP7 Is Associated with the Risk of Esophageal Squamous Cell Carcinoma in the Kazakh Population but Is Not Associated with HPV Infection. *PLoS ONE* **2017**, *12*, e0186319, doi:10.1371/journal.pone.0186319.
75. Lin, D.; Li, H.; Tan, W.; Miao, X.; Wang, L. Genetic Polymorphisms in Folate- Metabolizing Enzymes and Risk of Gastroesophageal Cancers: A Potential Nutrient-Gene Interaction in Cancer Development. *Forum Nutr* **2007**, *60*, 140–145, doi:10.1159/000107090.
76. Ji, A.; Wang, J.; Yang, J.; Wei, Z.; Lian, C.; Ma, L.; Chen, J.; Qin, X.; Wang, L.; Wei, W. Functional SNPs in Human C20orf54 Gene Influence Susceptibility to Esophageal Squamous Cell Carcinoma. *Asian Pacific journal of cancer prevention : APJCP* **2011**, *12*, 3207–3212.
77. Ni, B.; Chen, S.; Xie, H.; Ma, H. Functional Polymorphisms in Interleukin-23 Receptor and Susceptibility to Esophageal Squamous Cell Carcinoma in Chinese Population. *PLoS ONE* **2014**, *9*, e89111, doi:10.1371/journal.pone.0089111.
78. Yu, C.; Zhou, Y.; Miao, X.; Xiong, P.; Tan, W.; Lin, D. Functional Haplotypes in the Promoter of Matrix Metalloproteinase-2 Predict Risk of the Occurrence and Metastasis of Esophageal Cancer. *Cancer Res* **2004**, *64*, 7622–7628, doi:10.1158/0008-5472.Can-04-1521.
79. Pan, W.T.; Yang, J.Y.; Wei, J.Y.; Chen, H.W.; Ge, Y.X.; Zhang, J.F.; Wang, Z.Q.; Zhou, C.C.; Yuan, Q.P.; Zhou, L.Q.; et al. Functional BCL-2 Regulatory Genetic Variants Contribute to Susceptibility of Esophageal Squamous Cell Carcinoma. *Scientific Reports* **2015**, *5*, doi:10.1038/srep11833.
80. Lichun, Y.; Tang, C.M.C.; Wai Lau, K.; Lung, M.L. Frequent Loss of Heterozygosity on Chromosome 9 in Chinese Esophageal Squamous Cell Carcinomas. *Cancer Letters* **2004**, *203*, 71–77, doi:10.1016/j.canlet.2003.09.027.
81. Hu, N.; Huang, J.; Emmert-Buck, M.R.; Tang, Z.Z.; Roth, M.J.; Wang, C.; Dawsey, S.M.; Li, G.; Li, W.J.; Wang, Q.H.; et al. Frequent Inactivation of the TP53 Gene in Esophageal Squamous Cell Carcinoma from a High-Risk Population in China. *Clin Cancer Res* **2001**, *7*, 883–891.
82. Zhong, L.; Zhu, Z.Z.; Shen, Y.; Sun, G.; Zhao, X.; Zhang, S.; Yin, X.; Zhu, J.; Xu, Z.; Zhu, G. Frequent Germline Mutation in the BRCA2 Gene in Esophageal Squamous Cell Carcinoma Patients from a Low-Risk Chinese Population. *Asian Pacific journal of cancer prevention : APJCP* **2011**, *12*, 1771–1776.
83. Tan, W.; Chen, G.F.; Xing, D.Y.; Song, C.Y.; Kadlubar, F.F.; Lin, D.X. Frequency of CYP2A6 Gene Deletion and Its Relation to Risk of Lung and Esophageal Cancer in the Chinese Population. *Int J Cancer* **2001**, *95*, 96–101, doi:10.1002/1097-0215(20010320)95:2<96::aid-ijc1017>3.0.co;2-2.
84. Li, M.; Zhang, W.; Liu, C.; Shi, Y.; Tang, W.; Chen, S.; Gu, H.; Yin, J.; Zhang, Z.; Jiang, P. Forkhead Box A1 (FOXA1) Tagging Polymorphisms and Esophageal Cancer Risk in a Chinese Population: A Fine-Mapping Study. *Biomarkers* **2016**, *21*, 523–529, doi:10.3109/1354750X.2016.1160425.
85. Sang, Y.; Bo, L.; Gu, H.; Yang, W.; Chen, Y. Flap Endonuclease-1 Rs174538 G>A Polymorphisms Are Associated with the Risk of Esophageal Cancer in a Chinese Population. *Thorac Cancer* **2017**, *8*, 192–196, doi:10.1111/1759-7714.12422.
86. Zhao, H.; Zheng, L.; Li, X.; Wang, L. FasL Gene -844T/C Mutation of Esophageal Cancer in South China and Its Clinical Significance. *Scientific reports* **2014**, *4*, 3866, doi:10.1038/srep03866.
87. Wang, J.; Noffsinger, A.; Stemmermann, G.; Fenoglio-Preiser, C. Esophageal Squamous Cell Carcinomas Arising in Patients from a High-Risk Area of North China Lack an Association with Epstein-Barr Virus. *Cancer Epidemiol Biomarkers Prev* **1999**, *8*, 1111–1114.
88. Stolzenberg-Solomon, R.Z.; Qiao, Y.L.; Abnet, C.C.; Ratnasinghe, D.L.; Dawsey, S.M.; Dong, Z.W.; Taylor, P.R.; Mark, S.D. Esophageal and Gastric Cardia Cancer Risk and Folate- and Vitamin B 12-Related Polymorphisms in Linxian, China. *Cancer Epidemiology Biomarkers and Prevention* **2003**, *12*, 1222–1226.
89. Zhang, S.X.; Yang, S.; Xu, C.Q.; Hou, R.P.; Zhang, C.Z.; Xu, C.P. Equivocal Association of RAD51 Polymorphisms with Risk of Esophageal Squamous Cell Carcinoma in a Chinese Population. *Asian Pacific Journal of Cancer Prevention* **2014**, *15*, 763–767, doi:10.7314/APJCP.2014.15.2.763.
90. Sang, Y.; Shi, L.; Wu, Y.; Yang, W.; Gu, H.; Yin, J.; Yuan, L.; Liu, C.; Wang, X.; Shi, Y.; et al. Epiregulin Rs1460008 A>G Polymorphism Is Associated with Decreased Risk of Esophageal Squamous Cell Carcinoma in a Chinese Population. *International Journal of Clinical and Experimental Medicine* **2016**, *9*, 23685–23690.
91. Xiong, J.X.; Wang, Y.S.; Sheng, J.; Xiang, D.; Huang, T.X.; Tan, B.B.; Zeng, C.M.; Li, H.H.; Yang, J.; Meltzer, S.J.; et al. Epigenetic Alterations of a Novel Antioxidant Gene SLC22A3 Predispose Susceptible Individuals to Increased Risk of Esophageal Cancer. *International journal of biological sciences* **2018**, *14*, 1658–1668, doi:10.7150/ijbs.28482.
92. Ma, W.J.; Lv, G.D.; Zheng, S.T.; Huang, C.G.; Liu, Q.; Wang, X.; Lin, R.Y.; Sheyhidin, I.; Lu, X.M. DNA Polymorphism and Risk of Esophageal Squamous Cell Carcinoma in a Population of North Xinjiang, China. *World J Gastroenterol* **2010**, *16*, 641–647, doi:10.3748/wjg.v16.i5.641.

93. Suo, C.; Qing, T.; Liu, Z.; Yang, X.; Yuan, Z.; Yang, Y.J.; Fan, M.; Zhang, T.; Lu, M.; Jin, L.; et al. Differential Cumulative Risk of Genetic Polymorphisms in Familial and Nonfamilial Esophageal Squamous Cell Carcinoma. *Cancer Epidemiol Biomarkers Prev* **2019**, *28*, 2014–2021, doi:10.1158/1055-9965.Epi-19-0484.
94. Zhou, X.B.; Guo, M.; Quan, L.P.; Zhang, W.; Lu, Z.M.; Wang, Q.H.; Ke, Y.; Xu, N.Z. Detection of Human Papillomavirus in Chinese Esophageal Squamous Cell Carcinoma and Its Adjacent Normal Epithelium. *World Journal of Gastroenterology* **2003**, *9*, 1170–1173, doi:10.3748/wjg.v9.i6.1170.
95. Zhao, D.; Zhang, X.; Guo, Y.; Tan, W.; Lin, D. Cyclooxygenase-2 Gly587Arg Variant Is Associated with Differential Enzymatic Activity and Risk of Esophageal Squamous-Cell Carcinoma. *Molecular carcinogenesis* **2009**, *48*, 934-941, doi:10.1002/mc.20543.
96. Wu, Y.; Liu, X.; Hu, L.; Tao, H.; Guan, X.; Zhang, K.; Bai, Y.; Yang, K. Copy Number Loss of Variation_91720 in PIK3CA Predicts Risk of Esophageal Squamous Cell Carcinoma. *International journal of clinical and experimental pathology* **2015**, *8*, 14479–14485.
97. Yu, S.; Yuan, G.; Hu, F.; Li, Y.; Zhang, R.; Li, P.; Chen, Z.; Song, J. Contribution of ZBTB20 Polymorphisms to Esophageal Cancer Risk Among the Chinese Han Population. *Pharmacogenomics and Personalized Medicine* **2022**, *15*, 827–842, doi:10.2147/PGPM.S370963.
98. Zhang, B.; Xiao, Q.; Chen, H.; Zhou, T.; Yin, Y. Comparison of Tumor-Associated and Nontumor-Associated Esophageal Mucosa Microbiota in Patients with Esophageal Squamous Cell Carcinoma. *Medicine (United States)* **2022**, *101*, E30483, doi:10.1097/MD.0000000000030483.
99. Zhang, D.; Zhang, Q.; Zhou, L.; Huo, L.; Zhang, Y.; Shen, Z.; Zhu, Y. Comparison of Prevalence, Viral Load, Physical Status and Expression of Human Papillomavirus-16, -18 and -58 in Esophageal and Cervical Cancer: A Case-Control Study. *BMC Cancer* **2010**, *10*, 650, doi:10.1186/1471-2407-10-650.
100. Wang, J.B.; Dawsey, S.M.; Fan, J.H.; Freedman, N.D.; Tang, Z.Z.; Ding, T.; Hu, N.; Wang, L.M.; Wang, C.Y.; Su, H.; et al. Common Genetic Variants Related to Vitamin D Status Are Not Associated with Esophageal Squamous Cell Carcinoma Risk in China. *Cancer Epidemiol* **2015**, *39*, 157–159, doi:10.1016/j.canep.2014.12.013.
101. Hu, N.; Li, W.J.; Su, H.; Wang, C.; Goldstein, A.M.; Albert, P.S.; Emmert-Buck, M.R.; Kong, L.H.; Roth, M.J.; Dawsey, S.M.; et al. Common Genetic Variants of TP53 and BRCA2 in Esophageal Cancer Patients and Healthy Individuals from Low and High Risk Areas of Northern China. *Cancer Detection and Prevention* **2003**, *27*, 132–138, doi:10.1016/S0361-090X%2803%2900031-X.
102. Sung, H.; Yang, H.H.; Zhang, H.; Yang, Q.; Hu, N.; Tang, Z.Z.; Su, H.; Wang, L.; Wang, C.; Ding, T.; et al. Common Genetic Variants in Epigenetic Machinery Genes and Risk of Upper Gastrointestinal Cancers. *Int J Epidemiol* **2015**, *44*, 1341–1352, doi:10.1093/ije/dyv050.
103. Ko JM; Ning L; Zhao XK; Chai AWY; Lei LC; Choi SSA; Tao L; Law S; Kwong A; Lee NP; et al. BRCA2 Loss-of-Function Germline Mutations Are Associated with Esophageal Squamous Cell Carcinoma Risk in Chinese. *Int J Cancer* **2020**, *146*, 1042–1051, doi:10.1002/ijc.32619.
104. Zhu, J.; Wang, M.; Zhu, M.; He, J.; Wang, J.C.; Jin, L.; Wang, X.F.; Xiang, J.Q.; Wei, Q. Associations of PI3KR1 and MTOR Polymorphisms with Esophageal Squamous Cell Carcinoma Risk and Gene-Environment Interactions in Eastern Chinese Populations. *Scientific reports* **2015**, *5*, 8250, doi:10.1038/srep08250.
105. Yang, Y.; Zhou, Q.; Pan, H.; Wang, L.; Qian, C. Association Study of Map3k1 Snps and Risk Factors with Susceptibility to Esophageal Squamous Cell Carcinoma in a Chinese Population: A Case-Control Study. *Pharmacogenomics and Personalized Medicine* **2020**, *13*, 189–197, doi:10.2147/PGPM.S256230.
106. Li, D.; Diao, Y.; Fang, X.; Li, H. Association of the Polymorphisms of MTHFR C677T, VDR C352T, and MPO G463A with Risk for Esophageal Squamous Cell Dysplasia and Carcinoma. *Archives of Medical Research* **2008**, *39*, 594–600, doi:10.1016/j.arcmed.2008.04.006.
107. Zhu, J.; Liu, C.; Teng, X.; Yin, J.; Zheng, L.; Wang, L.; Tang, W.; Gu, H.; Gu, B.; Chen, L. Association of the Interleukin-18 Receptor 1 and Interleukin-18 Receptor Accessory Protein Polymorphisms with the Risk of Esophageal Cancer. *Biomedical Reports* **2016**, *4*, 227–235, doi:10.3892/br.2015.552.
108. Li, K.; Yin, X.; Yang, H.; Yang, J.; Zhao, J.; Xu, C.; Xu, H. Association of the Genetic Polymorphisms in XRCC6 and XRCC5 with the Risk of ESCC in a High-Incidence Region of North China. *Tumori* **2015**, *101*, 24–29, doi:10.5301/tj.5000206.
109. Liu, F.; Wei, W.Q.; Cormier, R.T.; Zhang, S.T.; Qiao, Y.L.; Li, X.Q.; Zhu, S.T.; Zhai, Y.C.; Peng, X.X.; Yan, Y.X.; et al. Association of Single Nucleotide Polymorphisms in the Prostaglandin-Endoperoxide Synthase 2 (PTGS2) and Phospholipase A(2) Group IIA (PLA2G2A) Genes with Susceptibility to Esophageal Squamous Cell Carcinoma. *Asian Pacific Journal of Cancer Prevention* **2014**, *15*, 1797–1802, doi:10.7314/apjcp.2014.15.4.1797.
110. Zhang, Y.G.; Wang, L.Z.; Wang, P.; Song, C.H.; Wang, K.J.; Zhang, J.Y.; Dai, L.P. Association of Single Nucleotide Polymorphisms in ERCC2 Gene and Their Haplotypes with Esophageal Squamous Cell Carcinoma. *Tumor Biology* **2014**, *35*, 4225–4231, doi:10.1007/s13277-013-1553-x.

111. Chen, W.; Yang, C.; Yang, L.; Qi, C.; Tian, S.; Han, Y.; Dou, Y.; Ma, Y.; Tian, D.; Zheng, Y. Association of Roasting Meat Intake with the Risk of Esophageal Squamous Cell Carcinoma of Kazakh Chinese via Affecting Promoter Methylation of P16 Gene. *Asia Pacific Journal of Clinical Nutrition* **2014**, *23*, 488–497, doi:10.6133/apjcn.2014.23.3.11.
112. Zhou, R.M.; Li, Y.; Wang, N.; Huang, X.; Cao, S.R.; Shan, B.E. Association of Programmed Death-1 Polymorphisms with the Risk and Prognosis of Esophageal Squamous Cell Carcinoma. *Cancer Genet* **2016**, *209*, 365–375, doi:10.1016/j.cancergen.2016.06.006.
113. Zhang, N.; Yu, C.; Wen, D.; Chen, J.; Ling, Y.; Terajima, K.; Akazawa, K.; Shan, B.; Wang, S. Association of Nitrogen Compounds in Drinking Water with Incidence of Esophageal Squamous Cell Carcinoma in Shexian, China. *Tohoku Journal of Experimental Medicine* **2012**, *226*, 11–17, doi:10.1620/tjem.226.11.
114. Zhang, J.; Schulz, W.A.; Li, Y.; Wang, R.; Zotz, R.; Wen, D.; Siegel, D.; Ross, D.; Gabbert, H.E.; Sarbia, M. Association of NAD(P)H: Quinone Oxidoreductase 1 (NQO1) C609T Polymorphism with Esophageal Squamous Cell Carcinoma in a German Caucasian and a Northern Chinese Population. *Carcinogenesis* **2003**, *24*, 905–909, doi:10.1093/carcin/bgg019.
115. Wu, J.; Zhang, L.; Luo, H.; Zhu, Z.; Zhang, C.; Hou, Y. Association of Matrix Metalloproteinases-9 Gene Polymorphisms with Genetic Susceptibility to Esophageal Squamous Cell Carcinoma. *DNA and Cell Biology* **2008**, *27*, 553–557, doi:10.1089/dna.2008.0732.
116. Zhang, J.; Li, Y.; Wang, R.; Wen, D.; Sarbia, M.; Kuang, G.; Wu, M.; Wei, L.; He, M.; Zhang, L.; et al. Association of Cyclin D1 (G870A) Polymorphism with Susceptibility to Esophageal and Gastric Cardiac Carcinoma in a Northern Chinese Population. *Int J Cancer* **2003**, *105*, 281–284, doi:10.1002/ijc.11067.
117. Wang, Y.; Wu, H.; Liu, Q.; Wang, C.; Fu, L.; Wang, H.; Zhu, W.; Fu, W.; Lv, Y.; Wang, S.; et al. Association of CHRNA5-A3-B4 Variation with Esophageal Squamous Cell Carcinoma Risk and Smoking Behaviors in a Chinese Population. *PLoS ONE* **2013**, *8*, e67664, doi:10.1371/journal.pone.0067664.
118. Zhang, X.F.; Wang, Y.M.; Ge, H.; Cao, Y.Y.; Chen, Z.F.; Wen, D.G.; Guo, W.; Wang, N.; Li, Y.; Zhang, J.H. Association of CDH1 Single Nucleotide Polymorphisms with Susceptibility to Esophageal Squamous Cell Carcinomas and Gastric Cardia Carcinomas. *Diseases of the Esophagus* **2008**, *21*, 21–29, doi:10.1111/j.1442-2050.2007.00724.x.
119. Yin, J.; Tang, W.; Long, T.; Pan, H.; Liu, J.; Lv, L.; Liu, C.; Shi, Y.; Zhu, J.; Sun, Y.; et al. Association of ALDH3B2 Gene Polymorphism and Risk Factors with Susceptibility of Esophageal Squamous Cell Carcinoma in a Chinese Population: A Case-Control Study Involving 2,358 Subjects. *Oncotarget* **2017**, *8*, 110153–110165, doi:10.18632/oncotarget.22656.
120. Zhou, L.; Zhang, X.; Li, Z.; Zhou, C.; Li, M.; Tang, X.; Lu, C.; Li, H.; Yuan, Q.; Yang, M. Association of a Genetic Variation in a MiR-191 Binding Site in MDM4 with Risk of Esophageal Squamous Cell Carcinoma. *PLoS ONE* **2013**, *8*, e64331, doi:10.1371/journal.pone.0064331.
121. Li, R.Z.; Sun, J. Association between XPD Gene Polymorphisms and Esophageal Squamous Cell Carcinoma. *Molecular Medicine Reports* **2013**, *7*, 674–678, doi:10.3892/mmr.2012.1215.
122. Cao, R.; Tang, W.; Chen, S. Association between BTLA Polymorphisms and Susceptibility to Esophageal Squamous Cell Carcinoma in the Chinese Population. *Journal of Clinical Laboratory Analysis* **2020**, *34*, e23221, doi:10.1002/jcla.23221.
123. Hu, N.; Su, H.; Li, W.J.; Giffen, C.; Goldstein, A.M.; Hu, Y.; Wang, C.; Roth, M.J.; Li, G.; Dawsey, S.M.; et al. Allelotyping of Esophageal Squamous-Cell Carcinoma on Chromosome 13 Defines Deletions Related to Family History. *Genes Chromosomes and Cancer* **2005**, *44*, 271–278, doi:10.1002/gcc.20242.
124. Li, G.; Hu, N.; Goldstein, A.M.; Tang, Z.Z.; Roth, M.J.; Wang, Q.H.; Dawsey, S.M.; Han, X.Y.; Ding, T.; Huang, J.; et al. Allelic Loss on Chromosome Bands 13q11-Q13 in Esophageal Squamous Cell Carcinoma. *Genes Chromosomes and Cancer* **2001**, *31*, 390–397, doi:10.1002/gcc.1158.
125. Hu, N.; Roth, M.J.; Emmert-Buck, M.R.; Tang, Z.Z.; Polymeropolous, M.; Wang, Q.H.; Goldstein, A.M.; Han, X.Y.; Dawsey, S.M.; Ding, T.; et al. Allelic Loss in Esophageal Squamous Cell Carcinoma Patients with and without Family History of Upper Gastrointestinal Tract Cancer. *Clinical Cancer Research* **1999**, *5*, 3476–3482.
126. Yang, X.; Zhang, T.; Yin, X.; Yuan, Z.; Chen, H.; Plymoth, A.; Jin, L.; Chen, X.; Lu, M.; Ye, W. Adult Height, Body Mass Index Change, and Body Shape Change in Relation to Esophageal Squamous Cell Carcinoma Risk: A Population-Based Case-Control Study in China. *Cancer Medicine* **2019**, *8*, 5769–5778, doi:10.1002/cam4.2444.
127. Song, X.; You, W.; Zhu, J.; Cui, X.; Hu, J.; Chen, Y.; Liu, W.; Wang, L.; Li, S.; Wei, Y.; et al. A Genetic Variant in MiRNA-219-1 Is Associated with Risk of Esophageal Squamous Cell Carcinoma in Chinese Kazakhs. *Disease Markers* **2015**, *2015*, 541531, doi:10.1155/2015/541531.
128. Song, Y.; Wang, Y.; Xu, L.; Ma, J.; Chen, E.; Zang, R.; Jia, W.; Tao, X.; Hu, L. A Genetic Variant in CHRNB3-CHRNA6 Increases Risk of Esophageal Squamous Cell Carcinoma in Chinese Populations. *Carcinogenesis* **2015**, *36*, 538–542, doi:10.1093/carcin/bgv019.

129. Guo, H.; Wang, K.; Xiong, G.; Hu, H.; Wang, D.; Xu, X.; Guan, X.; Yang, K.; Bai, Y. A Functional Varient in MicroRNA-146a Is Associated with Risk of Esophageal Squamous Cell Carcinoma in Chinese Han. *Fam Cancer* **2010**, *9*, 599–603, doi:10.1007/s10689-010-9370-5.
